# Comparing Antibody Interfaces to Inform Rational Design of New Antibody Formats

**DOI:** 10.3389/fmolb.2022.812750

**Published:** 2022-01-26

**Authors:** Monica L. Fernández-Quintero, Patrick K. Quoika, Florian S. Wedl, Clarissa A. Seidler, Katharina B. Kroell, Johannes R. Loeffler, Nancy D. Pomarici, Valentin J. Hoerschinger, Alexander Bujotzek, Guy Georges, Hubert Kettenberger, Klaus R. Liedl

**Affiliations:** ^1^ Department of General, Inorganic and Theoretical Chemistry, Center for Molecular Biosciences Innsbruck (CMBI), University of Innsbruck, Innsbruck, Austria; ^2^ Roche Pharma Research and Early Development, Large Molecule Research, Roche Innovation Center Munich, Penzberg, Germany

**Keywords:** antibodies, structure, interface characterization, interface dynamics, antibody design, bispecific antibody formats

## Abstract

As the current biotherapeutic market is dominated by antibodies, the design of different antibody formats, like bispecific antibodies and other new formats, represent a key component in advancing antibody therapy. When designing new formats, a targeted modulation of pairing preferences is key. Several existing approaches are successful, but expanding the repertoire of design possibilities would be desirable. Cognate immunoglobulin G antibodies depend on homodimerization of the fragment crystallizable regions of two identical heavy chains. By modifying the dimeric interface of the third constant domain (C_H_3-C_H_3), with different mutations on each domain, the engineered Fc fragments form rather heterodimers than homodimers. The first constant domain (C_H_1-C_L_) shares a very similar fold and interdomain orientation with the C_H_3-C_H_3 dimer. Thus, numerous well-established design efforts for C_H_3-C_H_3 interfaces, have also been applied to C_H_1-C_L_ dimers to reduce the number of mispairings in the Fabs. Given the high structural similarity of the C_H_3-C_H_3 and C_H_1-C_L_ domains we want to identify additional opportunities in comparing the differences and overlapping interaction profiles. Our vision is to facilitate a toolkit that allows for the interchangeable usage of different design tools from crosslinking the knowledge between these two interface types. As a starting point, here, we use classical molecular dynamics simulations to identify differences of the C_H_3-C_H_3 and C_H_1-C_L_ interfaces and already find unexpected features of these interfaces shedding new light on possible design variations. Apart from identifying clear differences between the similar C_H_3-C_H_3 and C_H_1-C_L_ dimers, we structurally characterize the effects of point-mutations in the C_H_3-C_H_3 interface on the respective dynamics and interface interaction patterns. Thus, this study has broad implications in the field of antibody engineering as it provides a structural and mechanistical understanding of antibody interfaces and thereby presents a crucial aspect for the design of bispecific antibodies.

## Introduction

Antibodies play a central role in the adaptive immune system, as they can recognize and neutralize foreign antigens (Chiu et al., 2019). In the last years, antibodies emerged as a new class of pharmaceuticals (Kaplon et al., 2020; Kaplon and Reichert, 2021), with over one hundred antibody-based drugs being marketed or pending approval.

**FIGURE 1:**
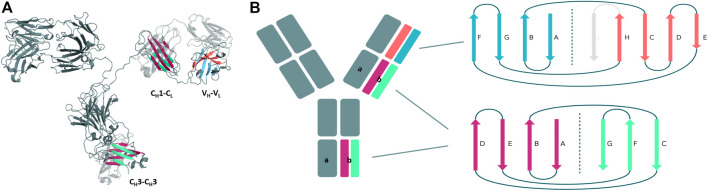
Structural and schematic representation of the modular anatomy of an antibody focusing on the discrete protein domains, which are characterized by the immunoglobulin fold. **(A)** Crystal structure of a whole IgG2 antibody (PDB: 1IGT) highlighting the different interface classes and their respective domain architecture. **(B)** Schematic depiction of the tertiary structure features, i.e., number and organization of the β-strands, for each of the individual antibody domains sharing the immunoglobulin fold. The C_H_3 and C_L_/C_H_1 domains do not only share a similar structure and topology but also contain the same number and arrangement of β-strands. The variable domains on the other hand differ in their number of β-strands and their architecture and are therefore color-coded differently.

Structurally, antibodies consist of two heavy and two light chains and have a unique modular anatomy facilitating their engineering and design (Davies and Chacko, 1993). The immunoglobulin heavy and light chains are composed of various discrete protein domains. Especially interesting is that these domains all have a similar folded structure, which is known as the immunoglobulin fold (Chiu et al., 2019). However, even though they share a similar fold, there are distinct structural differences between these domains (Figure 1). In general, antibodies can be divided into a crystallizable fragment (Fc) and two identical antigen-binding fragments (Fabs). The Fab can further be subdivided into constant (C_H_1-C_L_) and variable (V_H_-V_L_) domains (Davies and Chacko, 1993; Röthlisberger et al., 2005). The variable domains of the heavy and the light chain (V_H_ and V_L_) shape the antigen binding site and are responsible for antigen binding and recognition (Colman and Dixon, 1988; Addis et al., 2014; Fernández-Quintero et al., 2020c). The variable and the constant domains in the Fab are linked via a so-called switch region (Stanfield et al., 2006). The C_H_1-C_L_ heterodimer plays an essential role for antibody assembly and secretion in the cell (Adachi et al., 2003). Comparison of the V_H_-V_L_ and the C_H_1-C_L_ heterodimers revealed that the C_H_1-C_L_ heterodimer is more stable than the V_*H*_–V_*L*_ heterodimer (Röthlisberger et al., 2005). The individual C_H_1 domain is not stable in folded form and requires interactions with either the chaperone BiP or the C_L_ domain for folded state stability (Vanhove et al., 2001; Feige et al., 2014). The crystallizable fragment is composed of a C_H_2-C_H_2 and a C_H_3-C_H_3 homodimer (Teplyakov et al., 2013). The C_H_2-C_H_2 domain has no direct protein interactions in the interface as the interface is formed by glycans (Teplyakov et al., 2013). Thus, the C_H_2-C_H_2 domain differs from all other domains and consequently will not be discussed in this manuscript. The C_H_3 domains bind tightly with each other by hydrophobic interactions at the center, surrounded by salt bridges and thereby forming the foundation for the heavy chain dimer association (Teplyakov et al., 2013). Mutations in the C_H_3-C_H_3 interface have been shown to strongly influence the stability and the association of the two domains (Rose et al., 2013).

The concept of having an antibody with two different antigen binding sites was established more than 50 years ago by Nisonoff and co-workers and evolved alongside numerous advances and technical innovations in the field of antibody engineering, leading to more than 100 bispecific antibody (bsAb) formats known up to now (Nisonoff and Rivers, 1961; Fudenberg et al., 1964). BsAb formats expand the functionality of traditional antibodies by their ability to target effector cells to kill tumor cells, to enhance tissue specificity or to combine the antigen binding of two antibodies in a single molecule to simultaneously target two signaling pathways (Brinkmann and Kontermann, 2017; Sedykh et al., 2018). BsAbs can be assembled from different heavy and light chains. To suppress random assembly of different chains, resulting in various non-desired molecules, engineering efforts are required (Bönisch et al., 2017). A major breakthrough in the development of bsAb formats was the invention of the knobs-into-holes (KiH) technology for C_H_3-C_H_3 interfaces (Ridgway et al., 1996; Elliott et al., 2014). Precisely, advances like the KiH technology for C_H_3-C_H_3 interfaces represented a novel and effective design strategy for engineering heavy chain homodimers towards heterodimers, to reduce the risk of random assembly of different chains (Ridgway et al., 1996; Elliott et al., 2014; Kuglstatter et al., 2017). Thus, the idea of modifying the interfaces has motivated numerous studies to find variations of this approach by following a number of different strategies, such as alterations of the charge polarity in the interfaces compared to the homodimer, e.g., inverted charge interactions (DE-KK and DD-KK variants) (Ha et al., 2016; Moore et al., 2019). More recently, also KiH mutations in combination with charge inversions have been introduced into both Fab interfaces, C_H_1-C_L_ and V_H_-V_L_, enforcing the correct pairings of light chains with the corresponding heavy chains (Bönisch et al., 2017; Dillon et al., 2017; Regula et al., 2018).

In this study, we use classical molecular dynamics simulations to provide a systematic and extensive comparison of different antibody interfaces, which are in the spotlight of antibody engineering as they offer numerous design opportunities for bispecific antibody formats (Brinkmann and Kontermann, 2017; Sedykh et al., 2018). As C_H_1-C_L_ dimers are inherently heterodimers, we compare them with the homo-and-heterodimeric C_H_3-C_H_3 domains. We aim to identify different and overlapping interaction profiles of either the C_H_3-C_H_3 or C_H_1-C_L_ interfaces with the intention to crosslink the knowledge covering the two interfaces (Jost Lopez et al., 2020). Apart from that, we compare the interface flexibilities of C_H_3-C_H_3 or C_H_1-C_L_ domains and provide key determinants that contribute to the stability and their tendency to heterodimerize.

The investigated C_H_3-C_H_3 and C_H_1-C_L_ dimers and their respective PDB accession codes are summarized in Supplementary Table S1, covering a variety of different design strategies to enforce the formation of heterodimers. The Fabs to study the C_H_1-C_L_ dimers were chosen based on their availability of experimentally determined structure and stability data and their light chain isotypes. We also included in our dataset three antibody Fabs with mutations in the C_H_1-C_L_, which facilitate selective Fab assembly in combination with previously described KiH mutations for preferential heavy chain heterodimerization.

## Results

### Structural Architecture of the Investigated Antibody Interfaces

First, we introduce and structurally characterize different antibody interfaces and their respective architectures (Figure 1). All investigated antibody interfaces (23 Fab fragments and 23 C_H_3-C_H_3 domains), summarized in Supplementary Table S1, have been simulated for 1 µs with classical molecular dynamics simulations (extracting 10,000 frames) in explicit solvation to better understand and capture the variability of these interfaces. Figure 1 shows the comparison of the dimeric antibody interfaces in the antigen-binding fragment (V_H_-V_L_ and C_H_1-C_L_ domains) and in the third constant domain (C_H_3-C_H_3 domain). All of the presented interfaces share the same immunoglobulin fold, which is characterised by hydrogen bond interactions between the different β-strands. Additionally, we find that the C_H_1-C_L_ and C_H_3-C_H_3 domains have actually the same number of β-strands, i.e., a 3-stranded sheet packed against a 4-stranded sheet. Also, the relative orientation of the two monomers with respect to each other (approximately 90° observed in X-ray structures) is nearly identical between the C_H_3-C_H_3 and C_H_1-C_L_ dimers. Thus, the C_H_3-C_H_3 and C_H_1-C_L_ dimers share a very similar structure and fold. However, we observe structural differences in the overall architecture between the C_H_1-C_L_/C_H_3-C_H_3 and the V_H_-V_L_ domains, as the V_H_-V_L_ domains differ in their number of strands (9 β-strands arranged in two sheets of 4 and 5 strands), and in their relative orientation between the V_H_ and V_L_ monomers with respect to each other (approximately 50° observed in X-ray structures).

### Relative Interdomain Orientations of C_H_1-C_L_ and C_H_3-C_H_3 Domains

**FIGURE 2:**
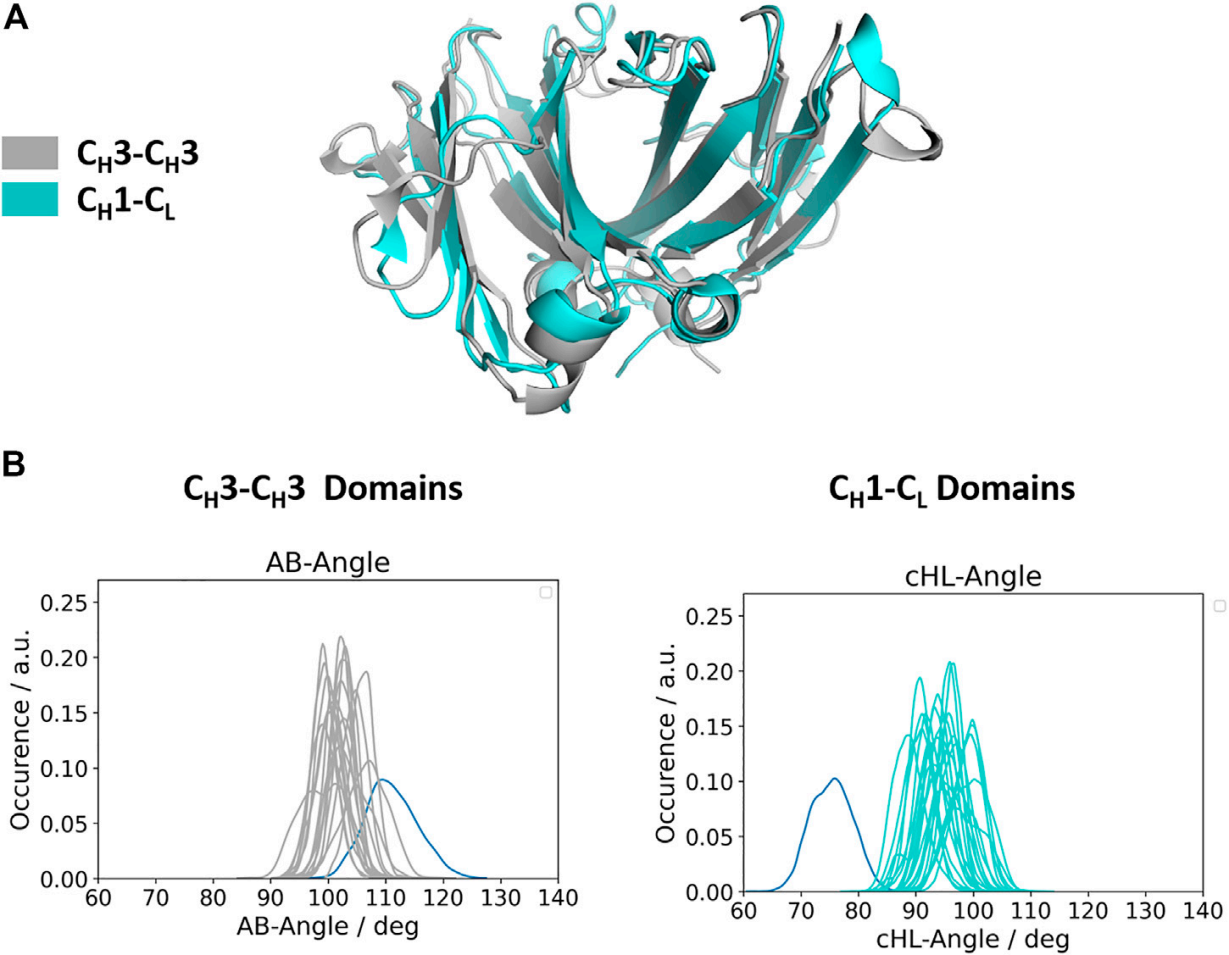
Comparison of the structurally highly similar C_H_3-C_H_3 and C_H_1-C_L_ domains. **(A)** Structural overlay of a C_H_3-C_H_3 (grey, PDB: 3AVE) and a C_H_1-C_L_ (cyan, PDB: 5I19) dimer illustrating their identical scaffold, despite having diverging loop structures. **(B)** Distributions of interdomain angles for all C_H_3-C_H_3 and C_H_1-C_L_ domains, respectively. These angles haven been calculated with the recently published OCD tool and show that the C_H_3-C_H_3 interfaces cover narrower angle ranges, while the C_H_1-C_L_, due to their higher number of sequence variations, reveal a larger spread. The highlighted distributions shown in blue correspond to the C_H_3-C_H_3 heterodimeric DE-KK variant (PDB: 5NSC) and to the λ-light chain antibody (PDB: 1NL0).

Apart from understanding the structural architecture, the dimeric interfaces are strongly influenced by the relative interdomain orientation and their respective dynamics. To calculate the interface movements, we used the well-established ABangle tool (Dunbar et al., 2013) and a recently presented python tool, called OCD tool (Hoerschinger et al., 2021), which both allow to calculate the interface orientations of different immunoglobulin like domains by defining five angles (one torsion angle (HL/AB)/four tilt angles (LC1, LC2, HC1, HC2/AC1, AC2, BC1, BC2) and one distance (dC). For the C_H_1-C_L_ domains we added the prefix c to the angle names (cHL, cLC1, cLC2, cHC1, cHC2, dC), as the C_H_1-C_L_ dimer forms the constant domain of the Fab fragment and to be able to distinguish them from the variable fragment (Fv) nomenclature. The detailed definition of these angles is presented in the methods section. Figure 2A shows a superimposition of the two dimers (C_H_1-C_L_ and C_H_3-C_H_3), highlighting the high structural similarity of the β-strands, while the loops on the other hand differ between the two dimers (Cα-RMSD 1.8Å). Figure 2B depicts the interdomain angle distributions of the relative interdomain orientations for all investigated C_H_1-C_L_ and C_H_3-C_H_3 simulations and shows significant overlaps in the interface angle (cHL/AB) distributions. However, the C_H_1-C_L_ shows a higher variability in the interface angle, which is reflected in broader angle distributions, compared to the C_H_3-C_H_3 dimer. Apart from the higher flexibility in the interdomain angle, we also find shifted C_H_1-C_L_ distributions towards lower cHL-Angle values. The torsion angle (cHL) of all C_H_1-C_L_ domains ranges from 65°–110°, while the torsion angle (AB angle) of all C_H_3-C_H_3 ranges from 85–125° (cHL angle, AB angle). The biggest difference in the relative interdomain orientations can be observed for the Fv torsion angles (HL angle), which range from 35°–80° (Supplementary Figure S1).

### Structural Characterization of the C_H_1-C_L_ and C_H_3-C_H_3 Interfaces

**FIGURE 3:**
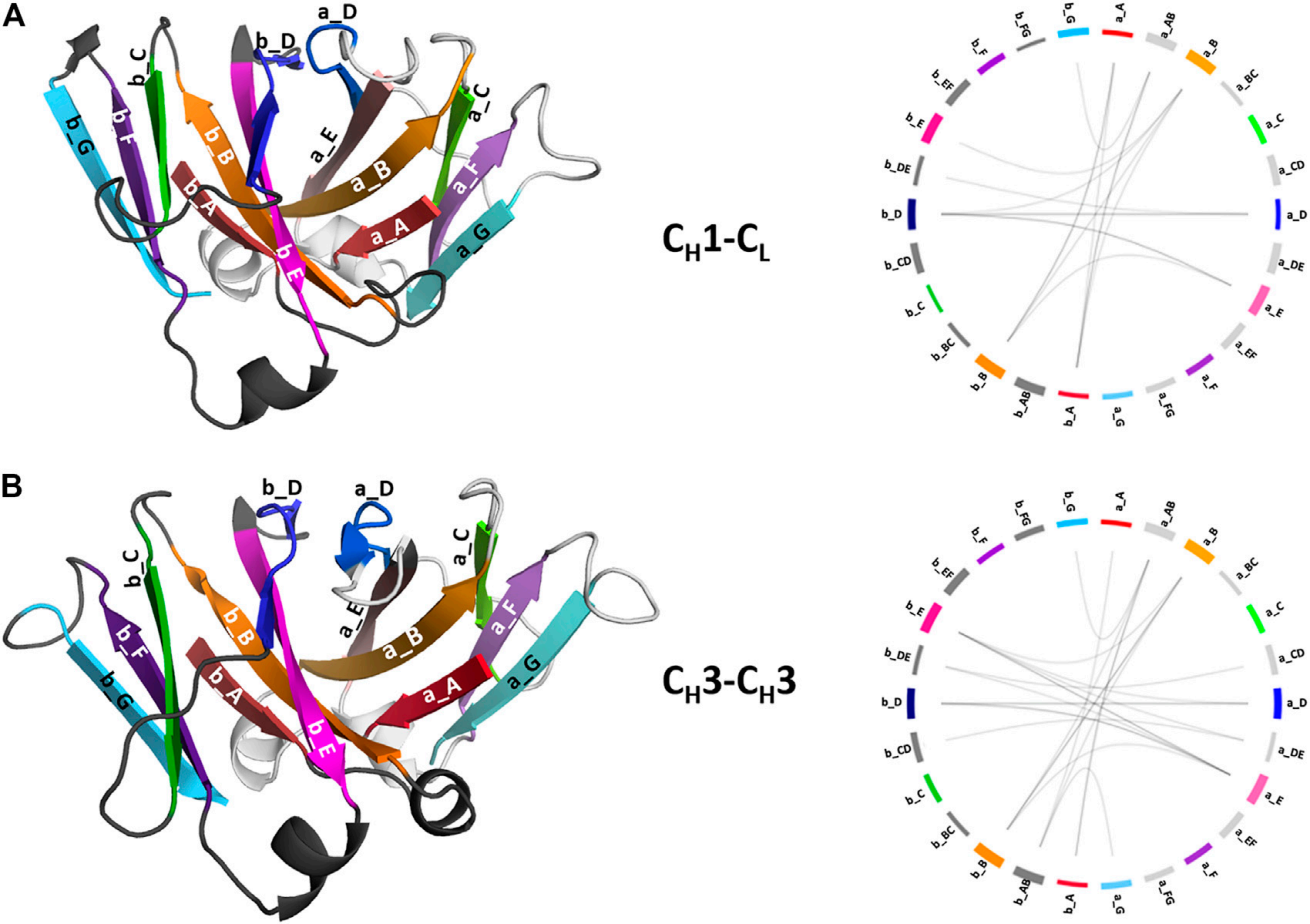
Structural representation of the C_H_3-C_H_3 and C_H_1-C_L_ domains including a coarse-grained contact analysis of the interactions contributing to the formation and stabilization of the domain interfaces. **(A)** Structure of C_H_1-C_L_ heterodimer (PDB: 5I17) color-coded and labeled according to the coarse-grained flareplots on the right showing the interdomain interactions present in the X-ray structure. We coarse grained residues belonging to the same loops or β-strands. **(B)** Structure of C_H_3-C_H_3 (PDB: 5DJ0) dimer color-coded and labeled according to the coarse-grained flareplots on the right, which illustrate the interdomain contacts present in the X-ray structure.

**FIGURE 4:**
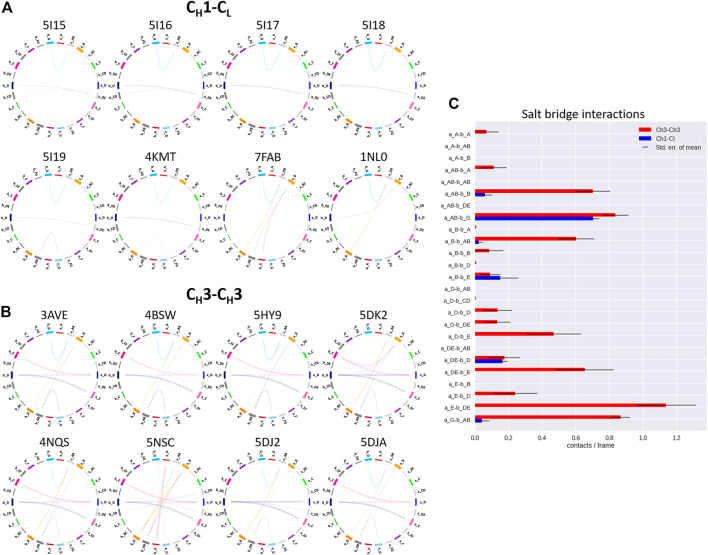
Exemplary coarse-grained flareplots showing the salt bridge interactions formed between the different interdomain β-strands and loops of both **(A)** C_H_1-C_L_ and **(B)** C_H_3-C_H_3 domains. **(C)** Bar plots quantitatively depicting differences in per strand/loop salt bridge interactions. We compare the two interface classes, i.e., C_H_1-C_L_ (blue) and C_H_3-C_H_3 (red). Thus, we show averages and standard errors of the mean of all investigated antibodies within the respective class.

**FIGURE 5:**
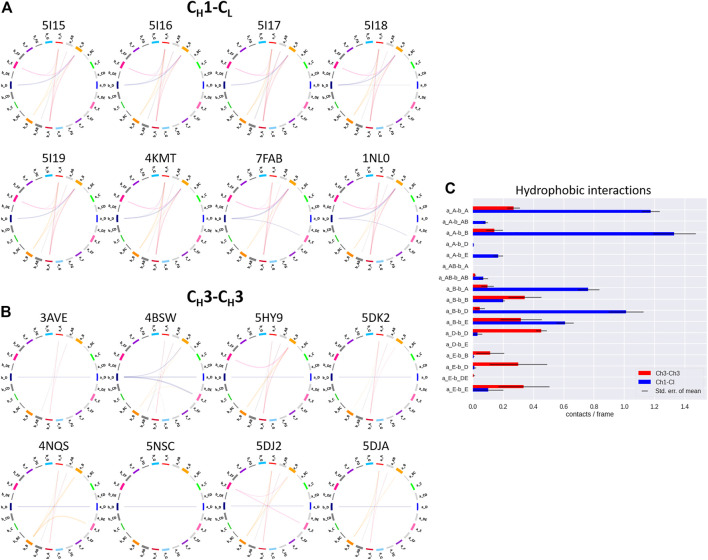
Exemplary coarse-grained flareplots showing the hydrophobic interactions formed between the different interdomain β-strands and loops of both **(A)** C_H_1-C_L_ and **(B)** C_H_3-C_H_3 domains. **(C)** Bar plots quantitatively depicting differences in per strand/loop hydrophobic interactions. We compare the two interface classes, i.e., C_H_1-C_L_ (blue) and C_H_3-C_H_3 (red). Thus, we show averages and standard errors of the mean of all investigated antibodies within the respective class.

To structurally characterize interactions in the C_H_1-C_L_ and C_H_3-C_H_3 interfaces, we use the GetContacts tool (Stanford University, adate), which calculates the interface contacts in a time-resolved way and depicts them with so-called flareplots (https://getcontacts.github.io/). To better visualize the comparison between the two interfaces we grouped the residues belonging to the same loops and β-strands to obtain coarse grained flareplots. This coarse-grained representation of the C_H_1-C_L_ and C_H_3-C_H_3 interfaces also allows having a better overview about the regions of these interfaces that actually form key interactions, which contribute to their structural integrity and to their stability. The β-strands are labelled with single letters, while the loops are tagged with a two-letter combination of the respective β-strands before and after the loop. To ease the comparison between C_H_1-C_L_ and C_H_3-C_H_3 we refer both to the C_L_ domain and one of the C_H_3 domains (the domain A) as “a” and to the C_H_1 domain and to the other C_H_3 domain (the domain B) as “b.” The thickness of the lines in the flareplots corresponds to the occurrence of a contact (ratio) over the whole simulation time (10,000 frames/simulation). A representative C_H_1-C_L_ and C_H_3-C_H_3 structure color-coded and labeled according to the flareplots (right) is depicted in Figure 3. The coarse-grained flareplots presented in Figure 3 show all interdomain contact patterns for both the C_H_1-C_L_ and C_H_3-C_H_3 interface. While the C_H_1-C_L_ and C_H_3-C_H_3 domains share common interaction patterns, we also investigated the type of interactions contributing to the formation of the respective interface. The flareplots shown in Supplementary Figure S2, Figures 4, 5 are just exemplary plots. The barplots on the right quantitatively summarize and compare the contacts observed for all investigated C_H_3-C_H_3 and C_H_1-C_L_ domains. Supplementary Figure S2 illustrates representative coarse-grained flareplots showing the interdomain hydrogen bond interactions of both the C_H_1-C_L_ and C_H_3-C_H_3 domains. While we find overlaps in the hydrogen bond interaction patterns for the C_H_3-C_H_3 and C_H_1-C_L_ interfaces (Supplementary Figures S2A,B), they differ substantially in number and occurrence of interdomain hydrogen bonds between C_H_1-C_L_ and C_H_3-C_H_3 domains, i.e., the C_H_3-C_H_3 domains form significantly more hydrogen bonds between the a_E – b_DE, a_DE – b_E, a_A – b_AB, a_B – b_E, a_B – b_B and a_G – b_AB loops/strands (Supplementary Figure S2).

In line with these observations, we find that the C_H_3-C_H_3 interfaces are strongly stabilized by salt bridges (Figure 4), while the C_H_1-C_L_ interfaces reveal substantially more hydrophobic interactions (Figure 5). Long-lasting salt bridge interactions (>60% of the simulation time) in the C_H_3-C_H_3 interfaces are formed by the a_E – b_DE, a_DE – b_E, a_D – b_E, a_B – b_AB, a_AB – b_B and a_G – b_AB loops/strands. Salt bridges between the a_AB – b_G and a_DE – b_D loops/strands are present in both C_H_1-C_L_ and C_H_3-C_H_3 domains (Figure 4). While the C_H_3-C_H_3 domains are characterized by a substantially higher number of charged interactions, the C_H_1-C_L_ domains are stabilized by hydrophobic interactions between the a_B – b_D, a_B – b_A, a_A – b_A, a_B – b_E and a_A – b_B strands. Even though the C_H_3-C_H_3 interface is strongly stabilized by salt bridge interactions, the hydrophobic interactions between the a_E – b_B, a_D – b_D, a_E – b_E and a_E – b_D strands (Figure 5C) are characteristic for the C_H_3-C_H_3 domains, compared to the C_H_1-C_L_ domains.

**FIGURE 6:**
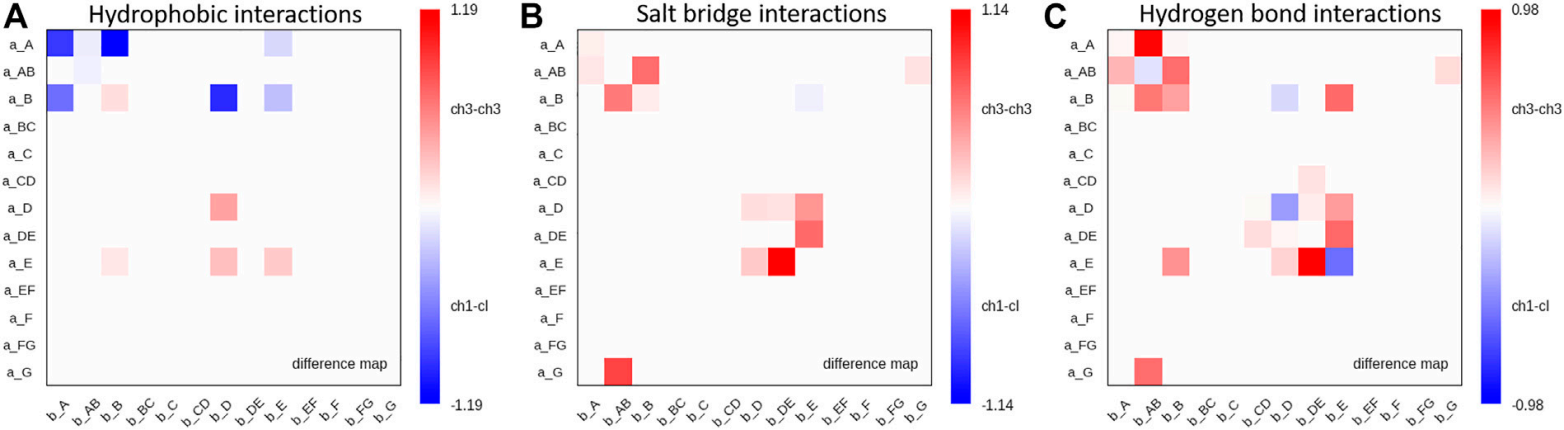
Maps depicting the differences in hydrophobic interactions, salt bridges and hydrogen bonds between C_H_3-C_H_3 and C_H_1-C_L_ domains. **(A)** Difference in hydrophobic interactions between all investigated C_H_3-C_H_3 and C_H_1-C_L_ domains (Supplementary Table S1) based on the previously defined coarse graining of the residues belonging to the same loops or β-strands. We normalized the colorbar according to the most frequent contact in either of the two interface classes. **(B)** Difference in salt bridge interactions between all investigated C_H_3-C_H_3 and C_H_1-C_L_ interfaces, showing the substantially higher number of salt bridge interactions dominating the C_H_3-C_H_3 interface. **(C)** Hydrogen bond difference maps for all investigated C_H_3-C_H_3 and C_H_1-C_L_ interfaces.

Moreover, we find interdomain van der Waals interaction patterns that are present in both the C_H_1-C_L_ and C_H_3-C_H_3 domains, e.g., interactions between a_D – b_D strands (Supplementary Figure S3). However, also substantial differences between C_H_1-C_L_ and C_H_3-C_H_3 domains can be identified for the interdomain van der Waals interactions, such as the interactions between the a_E – b_E strands and the a_A – b_AB strand/loop, which are dominantly present in C_H_3-C_H_3 domains and the a_E – b_D and a_A – b_B strands, which can be found more in C_H_1-C_L_ domains. Figure 6 illustrates contact maps depicting differences in the number and duration of hydrogen bond, salt bridge and hydrophobic interactions for all investigated antibody fragments. The color bar is normalized according to the most frequent contacts in either of the two interface classes.

Thus, Figure 6 summarizes the findings shown in Figures 4, 5 and Supplementary Figure S2, as it clearly displays the substantially higher number of hydrogen bond and salt bridge interactions for the C_H_3-C_H_3 domains, while the C_H_1-C_L_ interface is dominated by hydrophobic interactions. To quantify this difference even more, we calculated the electrostatic interface interaction energies for all investigated C_H_1-C_L_ and C_H_3-C_H_3 dimers (Supplementary Table S2). The strong difference in the type of interactions between the C_H_1-C_L_ and C_H_3-C_H_3 are even more pronounced in the electrostatic interface interaction energies, where we find significantly higher electrostatic interaction energies for the C_H_3-C_H_3 dimer, compared to the C_H_1-C_L_ domains.

Supplementary Figure S4 shows the comparison of three C_H_3-C_H_3 domains (Dengl et al., 2020) with three engineered C_H_1-C_L_ interfaces (Dillon et al., 2017), which were designed following similar heterodimerization strategies. The goal of redesigning the C_H_1/C_L_ interface was to reduce mispairings by having a stably paired C_H_1-C_L_ interface due to mutations that create incompatibilities towards the binding of wildtype C_H_1 or C_L_ domains (Dillon et al., 2017). Apart from inserting KiH mutations, the interface was redesigned by introducing charge mutations, which co-determine orthogonal heavy and light chain pairing preferences. The first two presented C_H_1-C_L_ domains (Supplementary Figures S4A,B) have newly introduced charge pairs and are therefore described as KE (C_H_1 S183K interacts with C_L_ V133E) and EK (C_H_1 S183E interacts with C_L_ V133K) variants (PDB accession codes: 5TDN and 5TDO, respectively). The third C_H_1-C_L_ interface (Supplementary Figure S4C) contains mutations at the edge of the interface at position C_L_ F116A and C_H_1 S181M, which introduce more flexibility. Additionally, KiH modifications are introduced at position C_H_1 F170S and C_L_ S176F (PDB accession code: 5TDP). Supplementary Figures S4A,B shows strong hydrogen bond networks for the KE and EK variants, especially between the a_E – b_E and a_B – b_E strands. Additionally, also strong salt bridge interactions can be observed for both the KE and EK variants between a_B – b_E strands, which cannot be observed in the third variant (Supplementary Figure S4C). Differences can also be observed in the hydrophobic contacts between the three engineered C_H_1-C_L_ variants. While hydrophobic contacts between a_A – b_A and a_A – b_B are present in all three variants, the third variant has long-lasting contacts between the a_E – b_E strands (Supplementary Figure S4C). Additionally, the two charge optimized C_H_1-C_L_ domains make strong hydrophobic interactions between the a_B – b_D and a_B – b_E strands (Supplementary Figures S4A,B). Comparing C_H_1-C_L_ variants with C_H_3-C_H_3 domains, we find that the EK and KE C_H_1-C_L_ variants (Supplementary Figures S4A,B) are able to form salt bridges between the a_B – b_E strands, which we only identified in C_H_3-C_H_3 domains before and not in other investigated C_H_1-C_L_ domains. The hydrophobic interactions of the KiH designed C_H_1-C_L_ domain (Supplementary Figure S4C) also show C_H_3-C_H_3 specific interactions between a_E – b_E strands, while the EK and KE variants show hydrophobic interaction patterns which are present in both C_H_3-C_H_3 and C_H_1-C_L_ domains. Panels d–f in Supplementary Figure S4 illustrate the interdomain interactions of three engineered C_H_3-C_H_3 variants, which are part of bispecific antibody matrices generated by Format Chain Exchange (FORCE), which enables the screening of the combinatorial format spaces (Dengl et al., 2020). These variants were originally designed by further modifying the 5HY9 KiH structure, which already differs from the 4NQS KiH structure by an additional intermolecular disulfide bridge.

**FIGURE 7:**
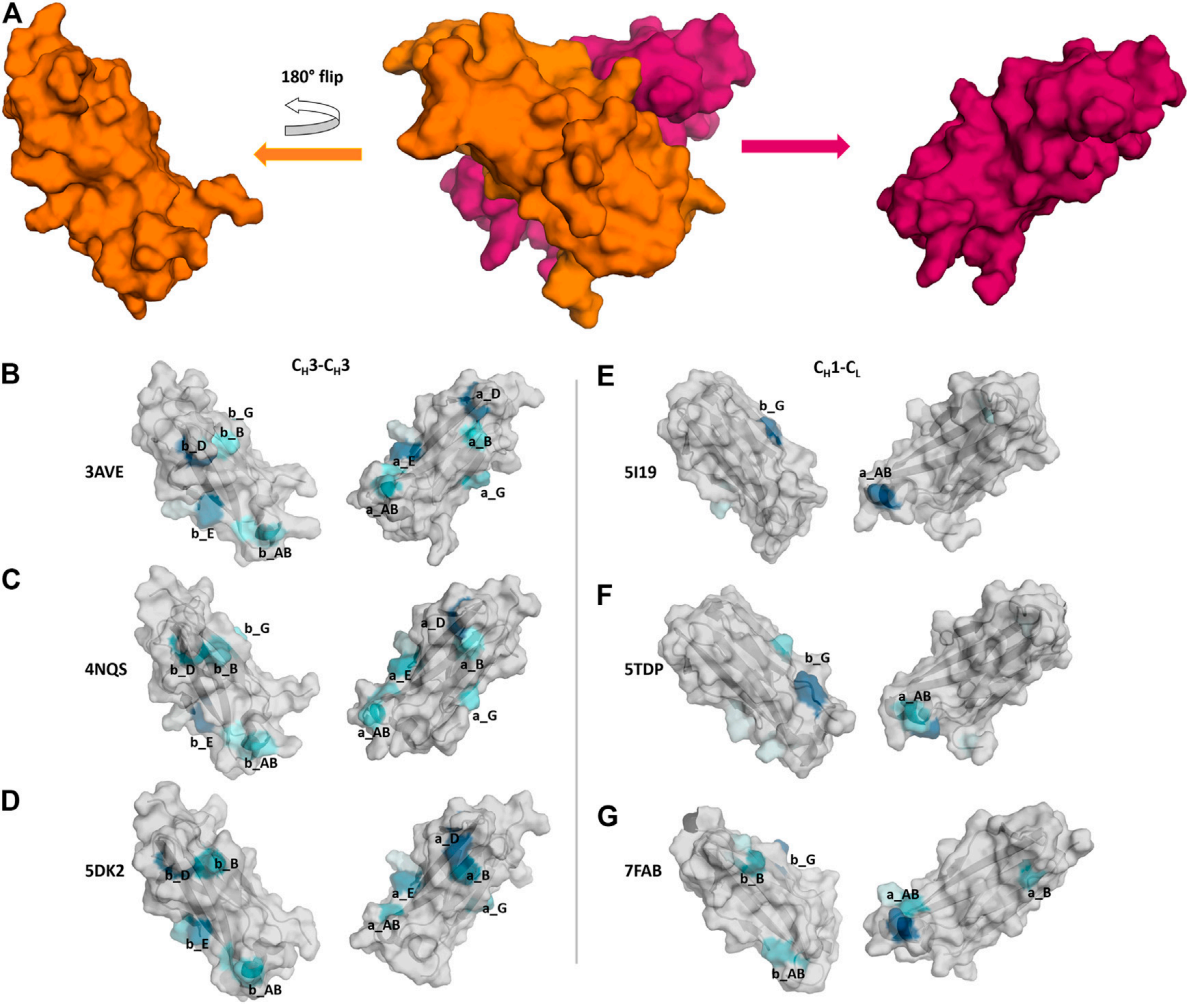
Comparison of C_H_3-C_H_3 and C_H_1-C_L_ interface interaction patterns by analyzing their salt bridge interdomain interactions. **(A)** Stepwise illustration of the workflow to obtain the “open-book” representation (PDB: 3AVE). **(B–G)** Each individual domain is gradually colored based on the number and duration of interdomain interactions. The color-gradient (grey to blue) corresponds to the number of interdomain salt bridges each residue is forming (the higher the number of contacts, the darker are the shades of blue).

In Figure 7 we show three exemplary C_H_1-C_L_ and three exemplary C_H_3-C_H_3 interfaces color-coded according to the number of interdomain salt bridge interactions. To facilitate the visualization of interface interactions, we flip the C_H_3 domain A and the C_L_ domain. In line with the results presented in Figure 6, we find that the C_H_3-C_H_3 interface is dominated by salt bridge interactions, while the C_H_1-C_L_ interface reveals a substantially lower number of ionic interactions, precisely the C_H_1-C_L_ reveals one characteristic salt bridge between loop a_AB and β-strand b_G.

### Structural C_H_3-C_H_3 Interface Characterization

**FIGURE 8:**
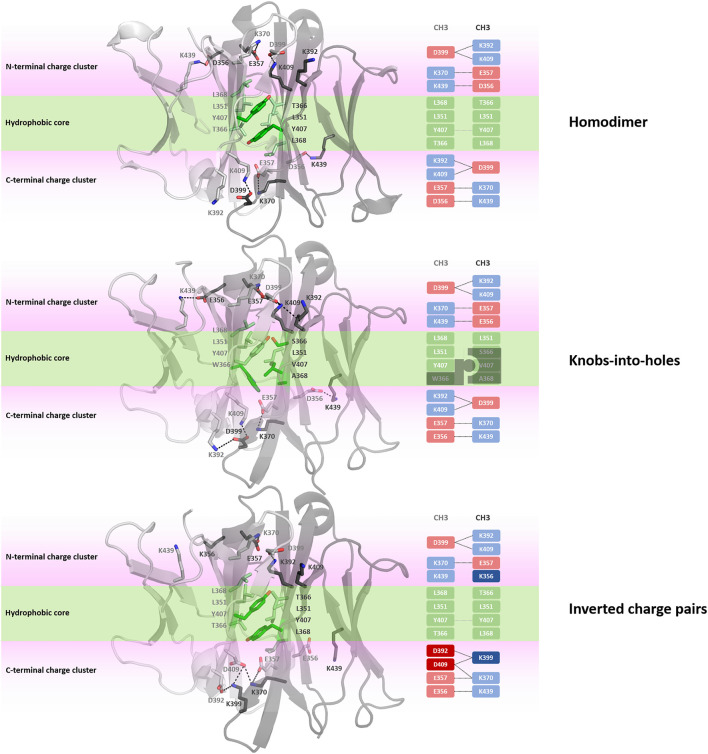
Interface interaction analysis of the homodimer and two heterodimers following different design strategies, i.e., knobs-into-holes (PDB: 4NQS) and charge inversion (PDB: 5DK2). The C_H_3-C_H_3 dimers consist of a hydrophobic core at the centre of the C_H_3-C_H_3 interface (illustrated in palegreen) and two highly charged regions (N-terminal charge cluster and C-terminal charge cluster), shown in light pink, that stabilize the interface.

Apart from identifying differences in interface interaction patterns between the structurally highly similar C_H_1-C_L_ and C_H_3-C_H_3 interfaces, we provide in Figure 8 an overview of the main interactions stabilizing the homo-and-heterodimeric C_H_3-C_H_3 interfaces (wildtype, KiH and charge inversion). Already from the panels in Figure 8 the unique and well-defined organization of the C_H_3-C_H_3 interface becomes apparent. Together with the hydrophobic core interactions (shown in green), various salt bridge interactions located at the N-terminal and C-terminal charge cluster (highlighted in pink) contribute to the stabilization of the dimeric interface. To characterize interactions and to identify residues that are critical for the interface formation, we analysed the investigated C_H_3-C_H_3 homo-and-heterodimer simulations in-detail. We find that the interactions in the core of the interface are particularly important for stabilization and formation of the dimer. One of these crucial interactions is the stacking interaction between residues Y407-Y407, which are present in all frames of the simulation in the variants with both interaction partners present (highlighted in Figure 8). We observe that especially mutations at the centre of the interface have a strong influence on the hydrophobic and salt bridge interaction network of the whole interface. One example would be the DE-KK variant (PDB accession code: 5NSC) (De Nardis et al., 2017), which introduces two ion pair interactions into the hydrophobic core by substituting L351D and L368E in one domain and L351K and T366K in the other. Even though these introduced residues strongly interact with each other, the mutations result in a change of the overall interdomain interaction patterns, which also differ from all other engineered variants. Particularly interesting is, that this DE-KK variant has the highest variability in the interdomain orientations (dC, AB, AC1, AC2, BC1, BC2) compared to all other investigated variants (Figure 2B). It also shows a slightly higher distance (dc) between the two domains and bigger variations in the tilt and bend angles, allowing also water molecules to interact with the N-terminal and C-terminal charge clusters. We also find similar results for the DD-KK variant (PDB accession code: 5DK2). The main difference between the DE-KK and the DD-KK variant is the location of the mutations. While the DE-KK disrupts the hydrophobic core interactions at the centre of the interface, the DD-KK variant introduces substitutions in the N-terminal charge cluster and C-terminal charge cluster. Introducing charge reversions in the charge clusters in this example results in an imbalance of positive and negative charges in the respective domains and, i.e., five negative charges in domain A, six positive charges in domain B. In particular the E356K mutation additionally results in a loss of a critical salt bridge interaction situated at the N-terminal charge cluster, which consequently shifts the interdomain tilt angles AC1 and BC1 and thereby increases the conformational variability in the interface. In line with these findings, we observe an increase in flexibility of the core interface residues for the KiH (PDB: 4NQS) and the charge inversion variants (PDB: 5DK2, 5NSC), which is reflected in higher root-mean-square-fluctuation (RMSF) values, compared to the homodimer (Supplementary Figure S7).

## Discussion

The idea of modifying antibody interfaces to reduce the risk of random assembly of different chains has motivated numerous studies to find variations of the proposed KiH approach, e.g., introducing charge pairs. Inverted charge interactions, instead of steric KiH interactions, were used for example for the design of the C_H_3-C_H_3 heterodimer DD-KK (K409D, K392D-D399K, E356K) variant (PDB accession code: 5DK2) (Ha et al., 2016). Also, the combination of the KiH interactions with the introduction of charge mutations have been presented in the C_H_3-C_H_3 heterodimer EW-RVT (K360E, K409W – Q347R, K399V, F405T) variant (PDB accession code: 4X98) (Choi et al., 2015).

One of the most frequent interactions situated in the centre of the homodimeric C_H_3-C_H_3 interface is the Y407-Y407 pi-stacking contact, residing in the central part of the E strands (Figure 8). (Dall’Acqua et al., 1998) Mutational studies confirmed the importance of these residues for the formation of the homodimeric interface. The salt bridge interactions at the N-terminal charge cluster and the C-terminal charge cluster (Figure 8) determine the characteristic interaction profile of the C_H_3-C_H_3 interface and substantially stabilize the dimer. The hydrophobic core in the homodimeric C_H_3-C_H_3 interface is formed by contacts between residues F405, L368, L351, Y407 and T366. These hydrophobic interactions are often modified following the KiH strategy (Ridgway et al., 1996; Elliott et al., 2014; Kuglstatter et al., 2017). The KiH variant (PDB accession codes: 4NQS, 5HY9, 5DI8) contains a knob in one C_H_3 domain (domain A) by mutating residue T366 to the bulkier amino acid tryptophane (Figure 8). Three other residues on the other C_H_3 domain (domain B) are also exchanged to smaller residues (T366S, Y407A, L368V) to ensure hydrophobic and steric complementarity. The orientation and position of the introduced tryptophane residue, also called “knob,” dominates the shape complementary between the two domains.

For the C_H_3-C_H_3 interfaces investigated in this study, we provide a sequence alignment showing the respective mutations including a classification of the underlying engineering strategies. To connect the sequence variations to our coarse-grained flareplots, we included our color-coded strand/loop definition in the alignment (Supplementary Figures S5, S6).

In our simulations of all different C_H_3-C_H_3 homo-and-heterodimers, we find that if both tyrosine residues are present, the pi-stacking interaction occurs in all frames of the simulation and contributes to stabilizing the interface. Additionally, Y407 forms a stabilizing and conserved hydrogen bond with T366, located in strand B, which occurs on average in 65% of the simulation time. Thus, as these Y407 residues form critical interactions, stabilizing the centre of the C_H_3-C_H_3 interface, mutating one of these residues can already prevent homodimerization (Ridgway et al., 1996; Von Kreudenstein et al., 2013). Additionally, we observe that introducing charge mutations/inversions at the hydrophobic core, can strongly influence the interface interaction network as shown for the DE-KK variant and result in a different interface formation, which can be accompanied by a decrease in stability. We find that this decrease in stability for the KiH (PDB: 4NQS) and the charge inversion variants (PDB: 5DK2, 5NSC), can result in a higher flexibility of the core interface residues, which is reflected in higher RMSF values (Supplementary Figure S7).

To compare the interaction patterns of the structurally highly similar C_H_1-C_L_ and C_H_3-C_H_3 interfaces, we calculate coarse-grained interdomain interaction maps, which are visualized as flareplots and quantified as barplots. When comparing different C_H_3-C_H_3 interfaces we find a highly conserved salt bridge between two glutamate residues (E356/E357) located in the a_AB loop with the lysine (K439) located in the b_G strand. These interactions can also be found in the C_H_1-C_L_ interfaces containing a λ light chain (PDB accession codes: 7FAB, 1NL0). Another critical conserved interdomain interaction among C_H_1-C_L_ domains can be found between the a_DE loop and the b_D strand, which is unique for kappa light chain antibodies. Especially for the salt bridges and hydrophobic interactions the patterns between κ and λ light chains differ the most (Figures 4A, 5A). Apart from the conserved contacts among all C_H_1-C_L_ interfaces, salt bridges are formed between the a_AB loops and b_B strands for the λ light chain antibodies. Interestingly, these salt bridges between a_AB loops and b_B strands are actually present in all considered C_H_3-C_H_3 domains (Figure 4B). Furthermore, an additional hydrophobic interaction can be found for the λ light chain antibodies between the a_E - b_D strands, which again can also be found in the C_H_3-C_H_3 interface (Figure 5C). Astonishingly, we observe in Figure 6 that the C_H_3 dimer is not only primarily stabilized by hydrophobic interactions but actually dominated by strong electrostatic interactions. Our observation, that the C_H_3-C_H_3 domains have a substantially higher number of salt bridges and hydrogen bonds, can also be explained by very frequently occurring interactions between residues D399-K409, D399-K392, E356-K439 and E357-K370, which surround the hydrophobic core. The high number of salt bridge interactions in the C_H_3-C_H_3 interface are also reflected in the electrostatic interaction energies, which are substantially higher compared to the C_H_1-C_L_ domains (Supplementary Table S2). However, there are high fluctuations in the electrostatic energies of the individual C_H_3-C_H_3 interfaces, which result from repairing salt bridge interactions between different residues. The C_H_1-C_L_ interface on the other hand is formed by mainly hydrophobic contacts.

The difference in electrostatic interaction energy is also reflected in the findings presented in Figure 7, which show a comparison of three C_H_3-C_H_3 and three C_H_1-C_L_ interfaces, illustrated as an “open-book” representation. The surfaces of the individual domains are color-coded according to the number of interdomain salt bridge interactions. We find substantial differences in the interface interaction patterns between the two interface classes. In particular, the C_H_1-C_L_ interface is dominated by one salt bridge between the a_AB loop and the b_G strand (Figure 7E). Figure 7F shows an engineered and mutated C_H_1-C_L_ interface, which contains mutations at the edge of the interface, which have been discussed to introduce more flexibility and indeed, we find more frequent switches in interdomain salt bridge interactions, which suggests a higher flexibility at the edge of the interface. In Figure 7G we depict interdomain salt bridge interactions of a C_H_1-C_L_ interface containing a λ light chain. In agreement with the results in Figure 4, we find more salt bridge interactions in C_H_1-C_L_ interface for λ light chain C_H_1-C_L_ domains and thus observe similar interaction patterns compared to the C_H_3-C_H_3 interfaces.

Apart from a detailed characterization of the C_H_3-C_H_3 and C_H_1-C_L_ interfaces, we also investigated the relative interdomain orientations during the simulations. In line with previous studies, we find that for the C_H_1-C_L_, as well as the C_H_3-C_H_3 domains, the majority of interdomain movements are surprisingly fast and can be captured in the low nanosecond timescale (Fernández-Quintero et al., 2020a; Fernández-Quintero et al., 2020b). Additionally, we observe for the investigated C_H_1-C_L_ domains (both λ and κ) left shifted cHL angle distributions towards lower cHL angles with a broader spread angle in the angle ranges, compared to the C_H_3-C_H_3 domains. For one λ light chain antibody (PDB accession code: 1NL0) we even observe a substantially shifted angle distribution towards lower cHL angle ranges. This higher variability in these cHL angle distributions is not surprising considering the higher number of sequence variations that occur in C_H_1-C_L_ domains, while the C_H_3-C_H_3 domains contain solely point mutations.

## Conclusion

In conclusion, we present a systematic characterization and a structural comparison of different C_H_1-C_L_ and C_H_3-C_H_3 domains. By using molecular dynamics simulations, we find substantial differences in interaction patterns of the structurally highly similar C_H_1-C_L_ and C_H_3-C_H_3 interfaces. While C_H_1-C_L_ interfaces are dominated by hydrophobic interactions, we find that the C_H_3-C_H_3 interfaces are stabilized by numerous salt bridge interactions surrounding the hydrophobic core. Furthermore, we provide quantitative contact maps comparing C_H_1-C_L_ and C_H_3-C_H_3 domains and highlighting which strands are key determinants for their structural integrity. Apart from the comparison, we also mechanistically discuss different C_H_3-C_H_3 interface engineering strategies, which provide an extensive understanding of the C_H_3-C_H_3 interfaces and thereby advance the design of bispecific antibodies.

## Methods

### Dataset

The investigated C_H_1-C_L_ and C_H_3-C_H_3 X-ray structures were chosen to have a representative set of antibodies covering various challenges in antibody engineering and design, as they differ in light chain types and follow different design strategies to reduce the risk of mispairings (Supplementary Table S1). (Ha et al., 2016; Teplyakov et al., 2016; Dillon et al., 2017; Dengl et al., 2020) 23 crystal structures of heterodimeric C_H_3 IgG1 mutants, as well as the corresponding wildtype were obtained from the PDB. The 23 mutants have been designed following different strategies: knobs-into-holes strategy, complementary electrostatic interactions, format chain exchange platform or by using Multistate Design (MSD), which is a computational sequence optimization tool.

Apart from the 23 C_H_3-C_H_3 domains, we also simulated 23 Fab crystal structures.

16 germline Fab crystal structures are from the same library (Teplyakov et al., 2016). We chose this dataset as it allows to systematically investigate the influence of different heavy and light chain pairings. The phage library is composed of 4 heavy chain germlines IGHV1-69 (H1-69), IGHV3-23 (H3-23), IGHV5-51 (H5-51) and IGHV3-53 (H5-53) and 4 light chain germlines (all κ) IGKV1-39 (L1-39), IGKV3-11 (L3-11), IGKV3-20 (L3-20) and IGKV4-1 (L4-1). These genes were selected based on the frequency of their use, their cognate canonical structures, which can recognize proteins and peptides and their ability to be expressed in bacteria. Additionally, we included three Fab fragments which were part of a study redesigning the Fab interfaces. Furthermore, we also investigated two λ light chain antibodies and two recently published DutaFab structures, which are characterized by their high stability and their ability to recognize two different antigens (Beckmann et al., 2021). Dual targeting (Duta) Fab molecules contain two independent and spatially separated binding sites within the CDR loops (H-side paratope and L-side paratope) that simultaneously allow to bind two target molecules at the same Fv.

### MD Simulation Protocol

All X-ray structures were prepared in MOE (Molecular Operating Environment, Montreal, QC, Canada: 2019) (Chemical Computing Group, 2020) using the Protonate 3D (Labute, 2009) tool. With the tleap tool of the Amber Tools20 package, we explicitly bonded all existing disulphide bridges (Supplementary Figure S8) and placed the Fab and C_H_3-C_H_3 structures into cubic water boxes of TIP3P(Jorgensen et al., 1983) water molecules with a minimum wall distance to the protein of 10 Å (El Hage et al., 2018; Gapsys and de Groot, 2019). Parameters for all antibody simulations were derived from the AMBER force field 14SB (Cornell et al., 1995; Maier et al., 2015). To neutralize the charges, we used uniform background charges (Darden et al., 1993; Salomon-Ferrer et al., 2013; Hub et al., 2014). Each system was carefully equilibrated using a multistep equilibration protocol (Wallnoefer et al., 2010; Wallnoefer et al., 2011).

Molecular dynamics simulations were performed using pmemd.cuda in an NpT ensemble to be as close to the experimental conditions as possible and to obtain the correct density distributions of both protein and water. Bonds involving hydrogen atoms were restrained by applying the SHAKE algorithm (Miyamoto and Kollman, 1992), allowing a timestep of 2.0 fs. Atmospheric pressure of the system was preserved by weak coupling to an external bath using the Berendsen algorithm (Berendsen et al., 1984). The Langevin thermostat was used to maintain the temperature at 300K during simulations (Adelman and Doll, 1976). The parameter file used to perform all MD simulations is provided at the end of the Supporting Information.

### Contacts

To calculate contacts of both C_H_1-C_L_ and C_H_3-C_H_3 interfaces we used the GetContacts software (Stanford University, adate). This tool can compute interactions within one protein structure, but also between different protein interfaces and allows to monitor the evolution of contacts during the simulation. The development of the contacts during a simulation can be visualized in so-called flareplots. For all available simulations (Supplementary Table S1) we calculated all different types of contacts, including hydrogen bonds (sidechain/sidechain, sidechain/backbone, backbone/backbone), salt bridges, hydrophobic and Van der Waals interactions. The contacts are determined based on the default geometrical criteria provided by GetContacts. To recognize interface patterns and to describe the dissociation mechanisms of both the C_H_1-C_L_ and C_H_3-C_H_3 domains, we coarse grained residues belonging to the same loops or β-strands. The secondary structure assignment has been performed with STRIDE (Frishman and Argos, 1995; Heinig and Frishman, 2004). To quantitively identify systematic differences in the interface interactions of the two interface classes, we evaluated the frequency of different interaction types. Thus, we counted contacts (for each type of interaction) of certain structural elements, e.g., salt bridges between the strand a_A and the loop b_AB. Furthermore, we calculated mean contact frequencies (contact per frame) in the simulations and averaged these frequencies within the interface classes and compared the results. In addition, we quantified the standard error of the mean of these contact frequencies within these classes. This comparison enabled us to find contacts, which, e.g., exist in all the C_H_1-C_L_ interfaces, but not in C_H_3-C_H_3 interfaces, or vice versa. Apart from visualizing and quantifying the contacts of both C_H_1-C_L_ and C_H_3-C_H_3 interfaces, we also calculated the linear interaction energies (LIE) by using the LIE tool implemented in cpptraj (Roe and Cheatham, 2013). We calculated the electrostatic interaction energies for all frames of each simulation (10,000 frames/simulation) and provided the simulation-averages of these interaction energies in Supplementary Table S2.

### Interdomain Orientation Calculations

While computational tools to fully characterize the Fv region of antibodies and TCRs are already available, no such tools were published for other immunoglobulin domain interfaces, such as the C_H_3-C_H_3 and the C_H_1-C_L_ interface (Dunbar et al., 2013). The OCD approach (Hoerschinger et al., 2021) creates a suitable coordinate system for the characterization of these interfaces for any user-provided reference structure. This allows a straight-forward analysis without the significant demands on previous structural knowledge. Using this tool, a reference coordinate system is created based on user-defined reference structures consisting of an atomic structure and two domain selections over these atoms. To this end, the reference structure for each domain is generated by considering a center axis linking the two centers of mass of the different domains, and the first principal axis P of inertia of each domain corresponding to the lowest eigenvalue of the inertia tensor. Each individual domain is aligned to the world coordinate system by aligning this principal axis to the z unit vector and the center axis as close as possible to the x unit vector, yielding a reference structure for each domain. To map the coordinate system onto a sample structure, the references are aligned to the sample and the alignment transformations are applied to the xyz unit vectors. The transformed z vectors (A1/B1) and y vectors (A2/B2) as well as the center axis are then used to calculate six orientational measures: Two tilt angles for each vector towards the center axis (AC1, AC2, BC1, BC2), the length of the center axis (dC) and a torsion angle (AB) between the two intersecting planes composed of A1, the centre axis and B1. To better visualize the relative interdomain orientations we performed the Gaussian kernel density estimation (KDE) on the HL angles, to obtain the probability density distributions. To calculate the KDE we used the recently published implementation of KDE in C++ (Kraml et al., 2021). We used 10,000 frames of each MD simulation (1µs) to calculate and plot the relative interdomain orientations.

### Relative V_H_ and V_L_ Orientations Using ABangle

ABangle is a computational tool (Dunbar et al., 2013; Bujotzek et al., 2015; Bujotzek et al., 2016; Fernández-Quintero et al., 2020b) to characterize the relative orientations between the antibody variable domains (V_H_ and V_L_) using six measurements (five angles and a distance). A plane is projected on each of the two variable domains. Between these two planes, a distance vector C is defined. The six measures are then two tilt angles between each plane (HC1, HC2, LC1, LC2) and a torsion angle (HL) between the two planes along the distance vector C (dC). The ABangle script can calculate these measures for an arbitrary Fv region by aligning the consensus structures to the found core set positions and fitting the planes and distance vector from this alignment. This online available tool was combined with an in‐house python script to reduce computational effort and to visualize our simulation data over time. The in‐house script makes use of ANARCI(Dunbar and Deane, 2016) for fast local annotation of the Fv region and pytraj from the AmberTools package (Case et al., 2020) for rapid trajectory processing.

## Data Availability

The original contributions presented in the study are included in the article/Supplementary Material, further inquiries can be directed to the corresponding author.
