# Corrigendum: Comparing Antibody Interfaces to Inform Rational Design of New Antibody Formats

**DOI:** 10.3389/fmolb.2022.864654

**Published:** 2022-02-15

**Authors:** Monica L. Fernández-Quintero, Patrick K. Quoika, Florian S. Wedl, Clarissa A. Seidler, Katharina B. Kroell, Johannes R. Loeffler, Nancy D. Pomarici, Valentin J. Hoerschinger, Alexander Bujotzek, Guy Georges, Hubert Kettenberger, Klaus R. Liedl

**Affiliations:** ^1^ Department of General, Inorganic and Theoretical Chemistry, Center for Molecular Biosciences Innsbruck (CMBI), University of Innsbruck, Innsbruck, Austria; ^2^ Roche Pharma Research and Early Development, Large Molecule Research, Roche Innovation Center Munich, Penzberg, Germany

**Keywords:** antibodies, structure, interface characterization, interface dynamics, antibody design, bispecific antibody formats

In the original article, we neglected to include the funder “European Union’s Horizon 2020 research and innovation programme under the Marie Skłodowska-Curie grant agreement No. 847476 to NP.”

The authors apologize for this error and state that this does not change the scientific conclusions of the article in any way. The original article has been updated.

