# Supplementary material for: Comparing Antibody Interfaces to Inform Rational Design of New Antibody Formats
Source: Front Mol Biosci. 2022 Jan 26;9:812750. doi: 10.3389/fmolb.2022.812750 (PMC8826573; doi:10.3389/fmolb.2022.812750)
Supplement: Supplementary file 1 [file DataSheet1.docx]

SI Table S1: Overview of all investigated C_H_3-C_H_3 and C_H_1-C_L_ interfaces sorted by their design strategies.

| **C_H_3-C_H_3-Domains** | | **C_H_1-C_L_ Domains** | |
| --- | --- | --- | --- |
| PDB | Features | PDB | Features |
| 3AVE | Wildtype | 5I15 | Different Heavy and Light Chain Pairings |
| 5DK2 | Charged Interactions | 5I16 |  |
| 5NSC |  | 5I17 |  |
| 4X98 |  | 5I18 |  |
| 5DJD | Computational Round 1:  T366/Y409 | 5I19 |  |
| 5DJ6 |  | 5I1A |  |
| 5DJA |  | 5I1C |  |
| 5DJ8 | Computational Round 1:  T366/Y409 + D399/K409 | 5I1D |  |
| 5DJZ |  | 5I1E |  |
| 5DJ2 |  | 5I1G |  |
| 5DJC |  | 5I1H |  |
| 5DJX | Computational Round 1: K370 | 5I1I |  |
| 5DJ0 |  | 4KMT |  |
| 5DJY | Computational Round 2 | 5I1L |  |
| 5DK0 |  | 1NL0 | Lambda Light Chain |
| 4NQS | Knop-into-hole | 7FAB |  |
| 4BSW |  | 1BBD | Kappa Light Chain |
| 4BSV |  | 1DBA |  |
| 5HY9 |  | 1PLG |  |
| 5TPS |  | 5TDN | Charged Interactions |
| 6YTB | FORCE | 5TDO |  |
| 6YT7 |  | 5TDP | Knob-into-hole |
| 6YSC |  | 6T9D | DutaFab |

|  | **C_H_3-C_H_3-Domains** | |  | **C_H_1-C_L_ Domains** | |
| --- | --- | --- | --- | --- | --- |
| PDB | Electrostatic Interaction Energy / kcal/mol | Standard deviation / kcal/mol | PDB | Electrostatic Interaction Energy / kcal/mol | Standard deviation / kcal/mol |
| 3AVE | -437.18 | 70.35 | 5I1C | -149.19 | 60.02 |
| 4BSV | -368.42 | 81.68 | 5I19 | -222.91 | 54.45 |
| 4BSW | -354.52 | 70.90 | 5I18 | -70.73 | 27.13 |
| 4NQS | -413.84 | 80.52 | 5I17 | -69.49 | 29.53 |
| 4X98 | -460.46 | 75.21 | 5I16 | -84.30 | 35.77 |
| 5DJ0 | -431.43 | 75.10 | 5I1D | -199.89 | 47.71 |
| 5DJ2 | -364.76 | 80.17 | 5I1E | -125.85 | 39.75 |
| 5DJ6 | -399.33 | 71.68 | 5I1H | -112.18 | 49.88 |
| 5DJ8 | -291.27 | 75.56 | 5I1I | -138.46 | 37.65 |
| 5DJA | -409.53 | 78.05 | 4KMT | -164.64 | 46.62 |
| 5DJC | -429.13 | 81.47 | 5I1L | -183.85 | 53.42 |
| 5DJD | -409.88 | 75.22 | 5I1G | -129.41 | 34.65 |
| 5DJX | -550.63 | 88.07 | 1DBA | -133.72 | 36.92 |
| 5DJY | -371.98 | 81.96 | 1NL0 | -257.32 | 93.83 |
| 5DJZ | -328.09 | 93.37 | 5I1A | -119.76 | 40.73 |
| 5DK0 | -348.75 | 84.36 | 1PLG | -90.53 | 38.89 |
| 5DK2 | -525.36 | 88.81 | 1BBD | -178.22 | 56.42 |
| 5NSC | -471.014 | 91.49 | 5I15 | -126.14 | 42.54 |
| 4X99 | -481.02 | 80.85 | 6T9D | -141.14 | 50.62 |
| 6YTB | -450.93 | 77.97 | 7FAB | -209.69 | 50.23 |
| 6YT7 | -413,63 | 67.38 | 5TDN | -257.51 | 53.83 |
| 6YSC | -247.59 | 77.83 | 5TDO | -191.81 | 42.69 |
| 5HY9 | -393.77 | 66.38 | 5TDP | -153.67 | 43.71 |

SI Table S2: Simulation-average of the electrostatic interaction energies for the investigated C_H_3-C_H_3 and C_H_1-C_L_ interfaces calculated for 1µs of MD simulations.


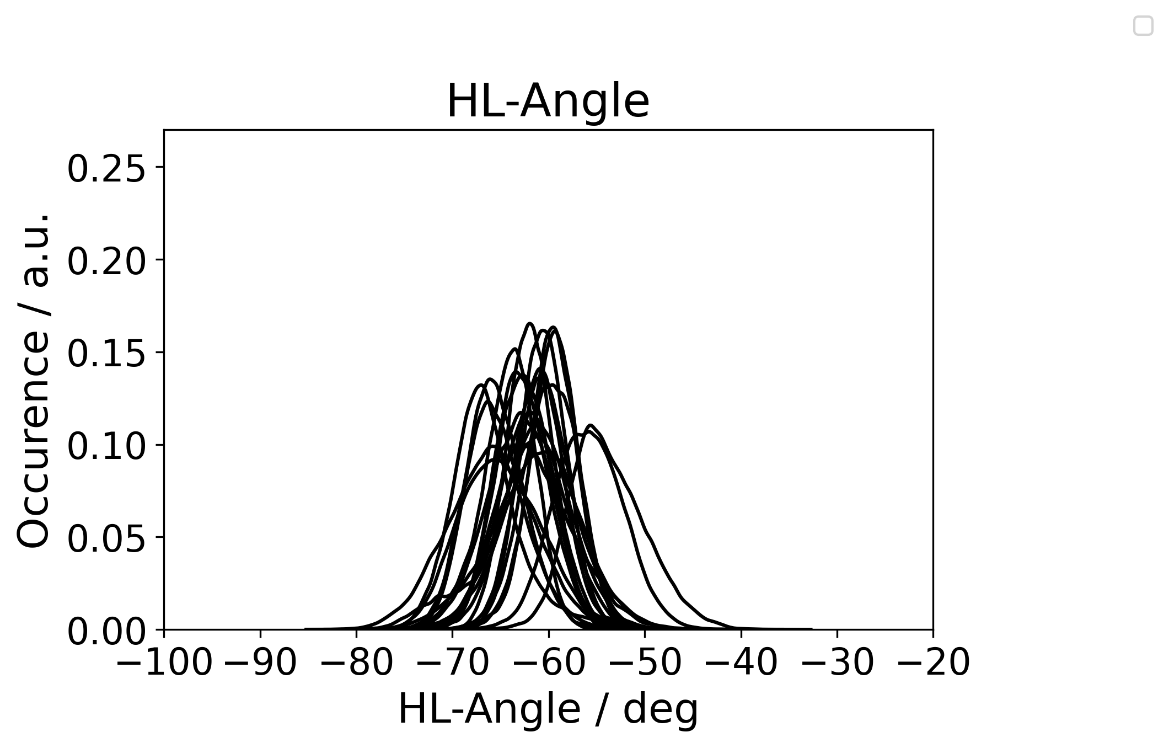


SI Figure S1: Probability density distributions of the interdomain dynamics for all V_H_-V_L_ domains, calculated with the well established ABangle tool. These distributions show that the V_H_-V_L_ interfaces cover substantially different angle ranges, compared to the structurally similar C_H_1-C_L_ and C_H_3-C_H_3 domains.


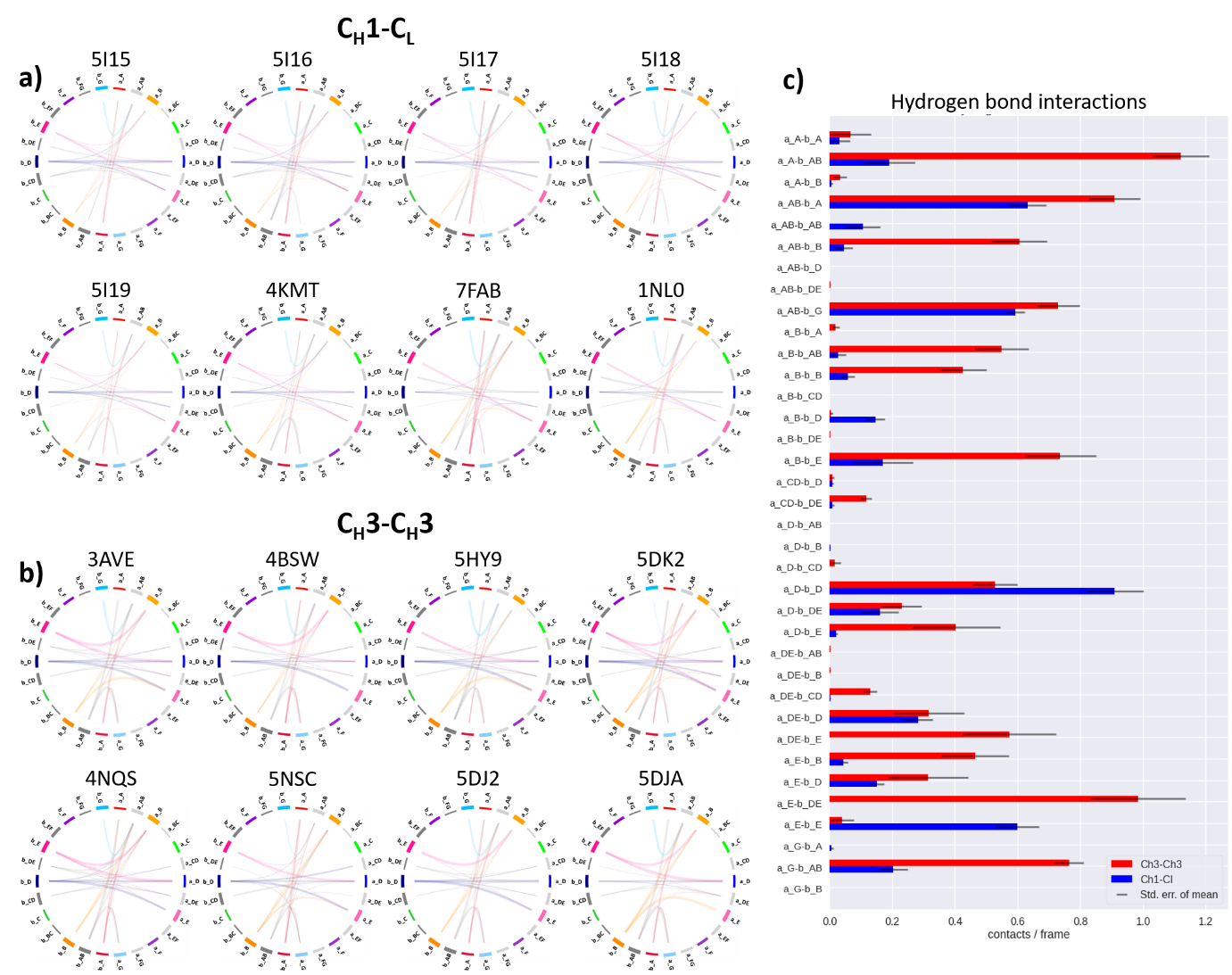


SI Figure S2: Exemplary coarse-grained flareplots showing the hydrogen bond interactions formed between the different interdomain β-strands and loops of both a) C_H_1-C_L_ and b) C_H_3-C_H_3 domains. c) Bar plots quantitatively depicting differences in per strand/loop hydrogen bond interactions. We compare the two interface classes, i.e., C_H_1-C_L_ (blue) and C_H_3-C_H_3 (red). Thus, we show averages and standard errors of the mean of all investigated antibodies within the respective class.


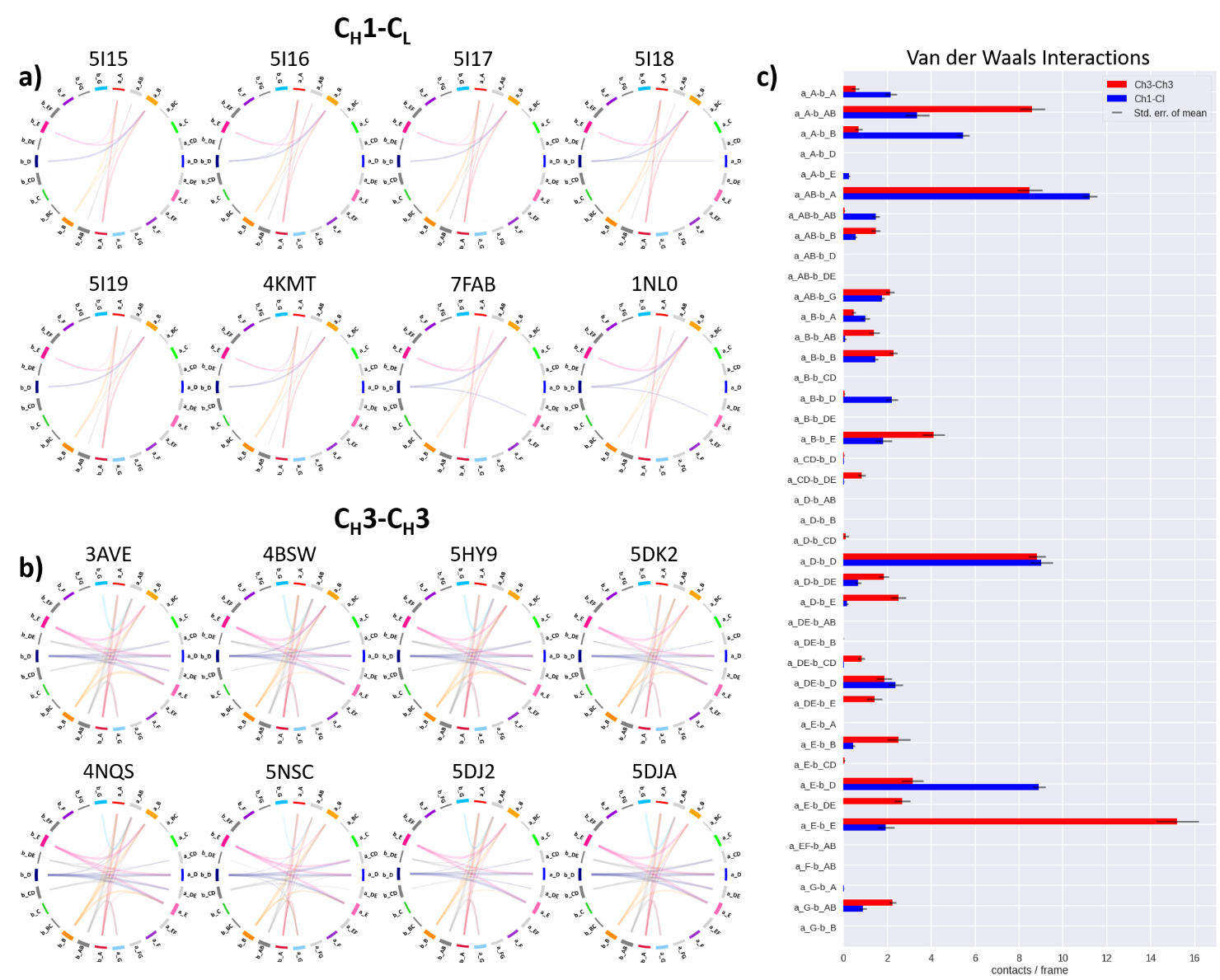


SI Figure S3: Exemplary coarse-grained flareplots showing the Van der Waals interactions formed between different β-strands and loops of both a) C_H_1-C_L_ and b) C_H_3-C_H_3 domains. c) Bar plots quantitatively depicting differences in per strand/loop Van der Waals interactions. We compare the two interface classes, i.e., C_H_1-C_L_ (blue) and C_H_3-C_H_3 (red). Thus, we show averages and standard errors of the mean of all investigated antibodies within the respective class.


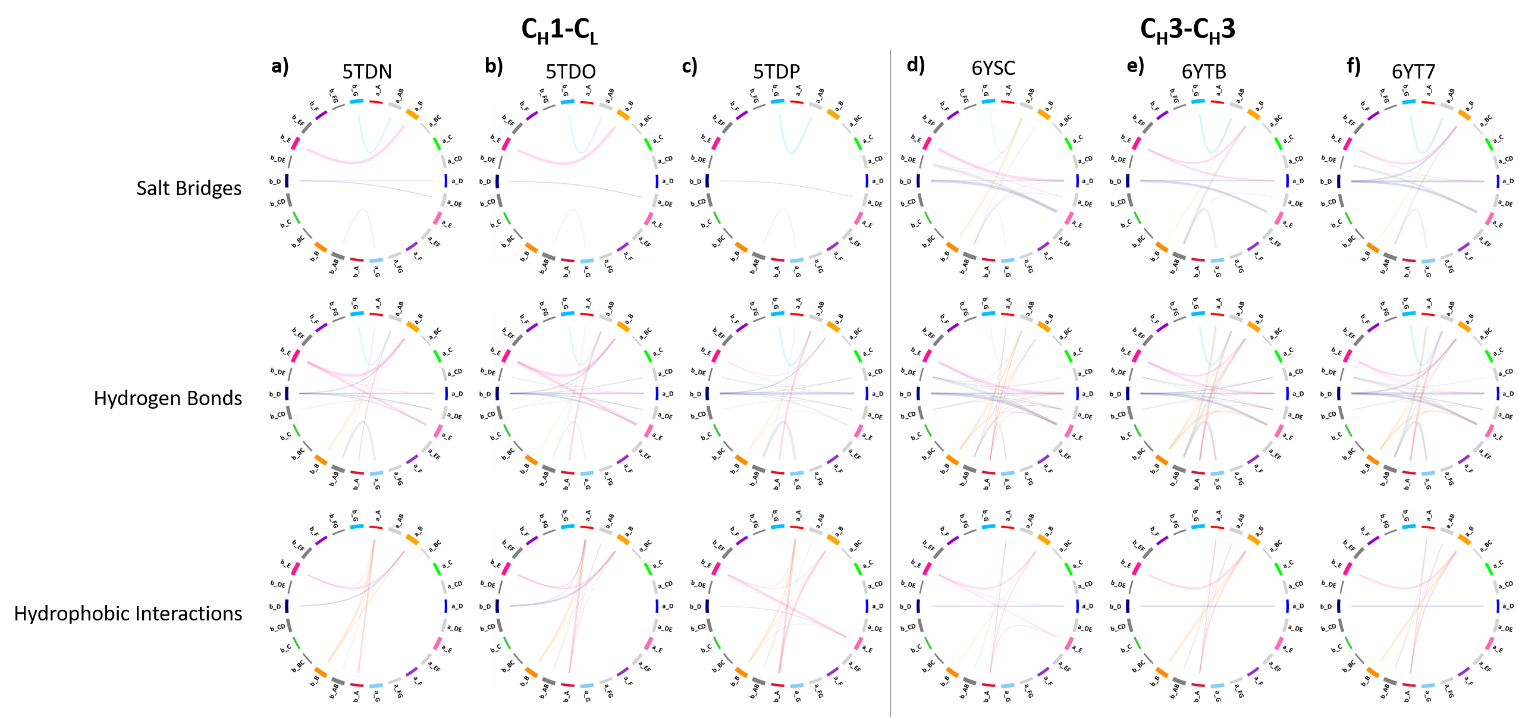


SI Figure S4: Coarse grained flareplots for each three engineered C_H_1-C_L_ and b) C_H_3-C_H_3 domains. The flareplots are split up into salt bridge interactions, hydrogen bond interactions and hydrophobic interactions.


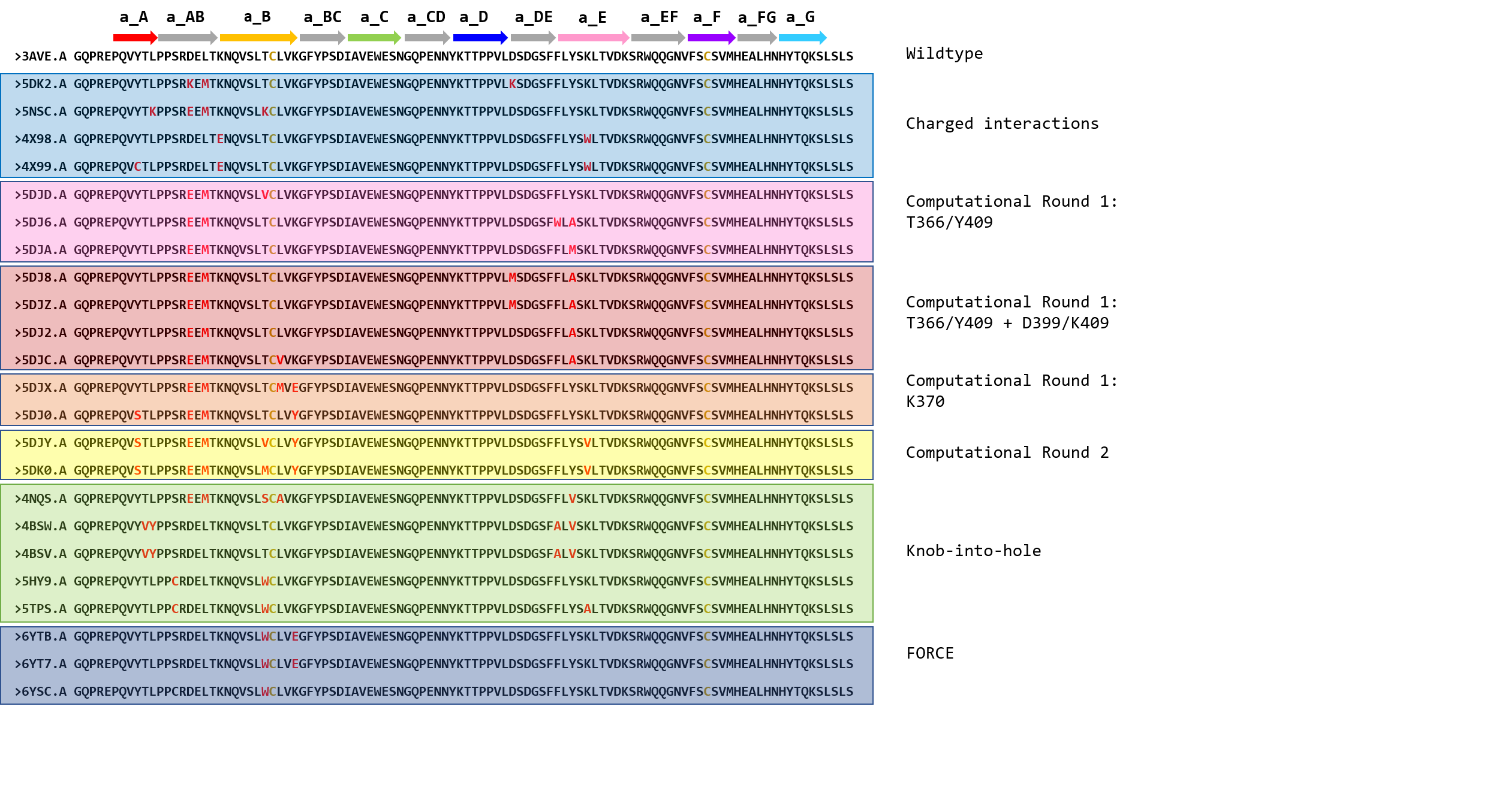


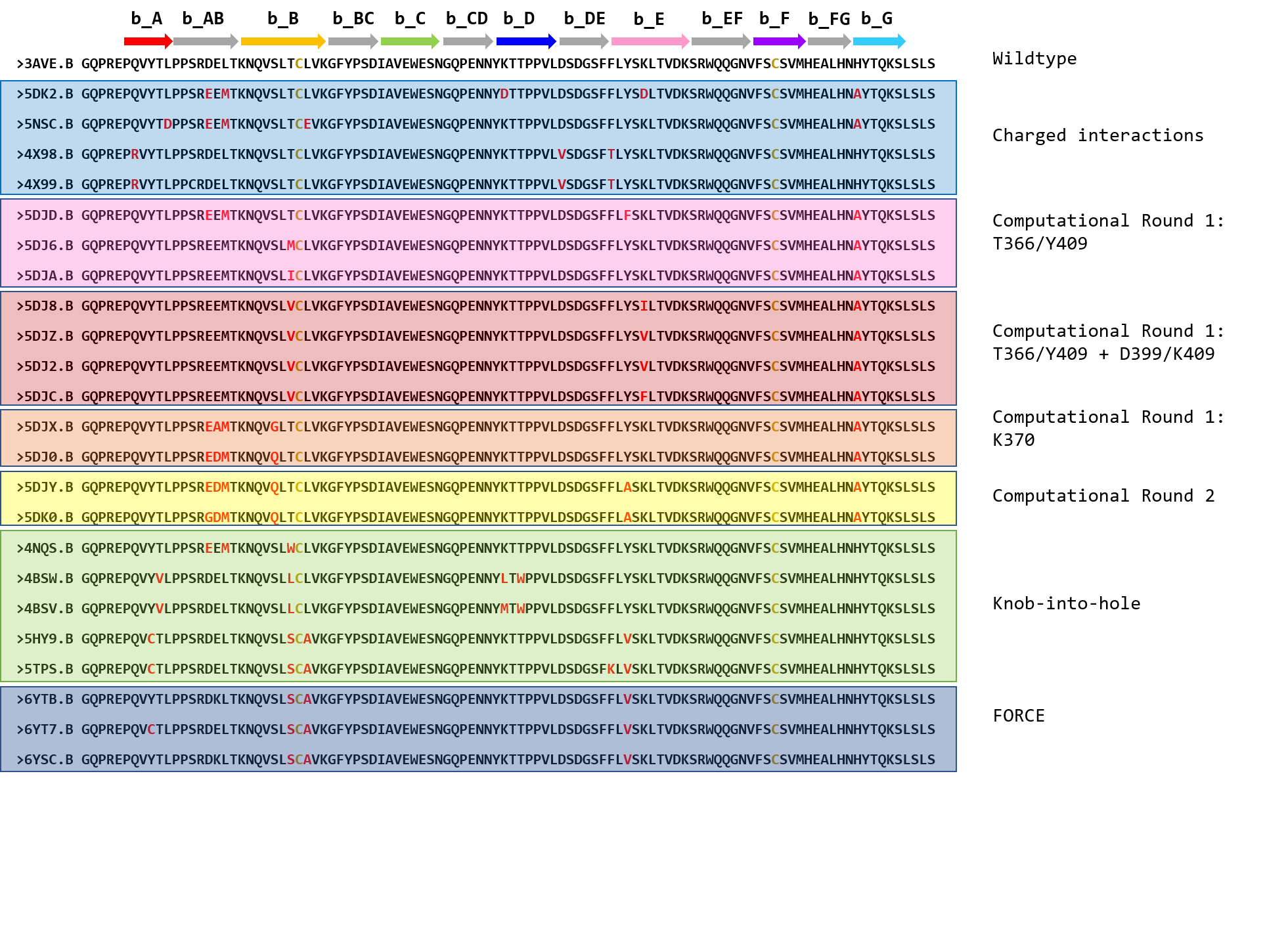
SI Figure S5: Sequence alignment of all investigated C_H_3-C_H_3 dimers. As reference sequence the homodimer (PDB code: 3AVE) has been used. The color-coding corresponds to the different design strategies that have been followed. In red the mutations are highlighted.
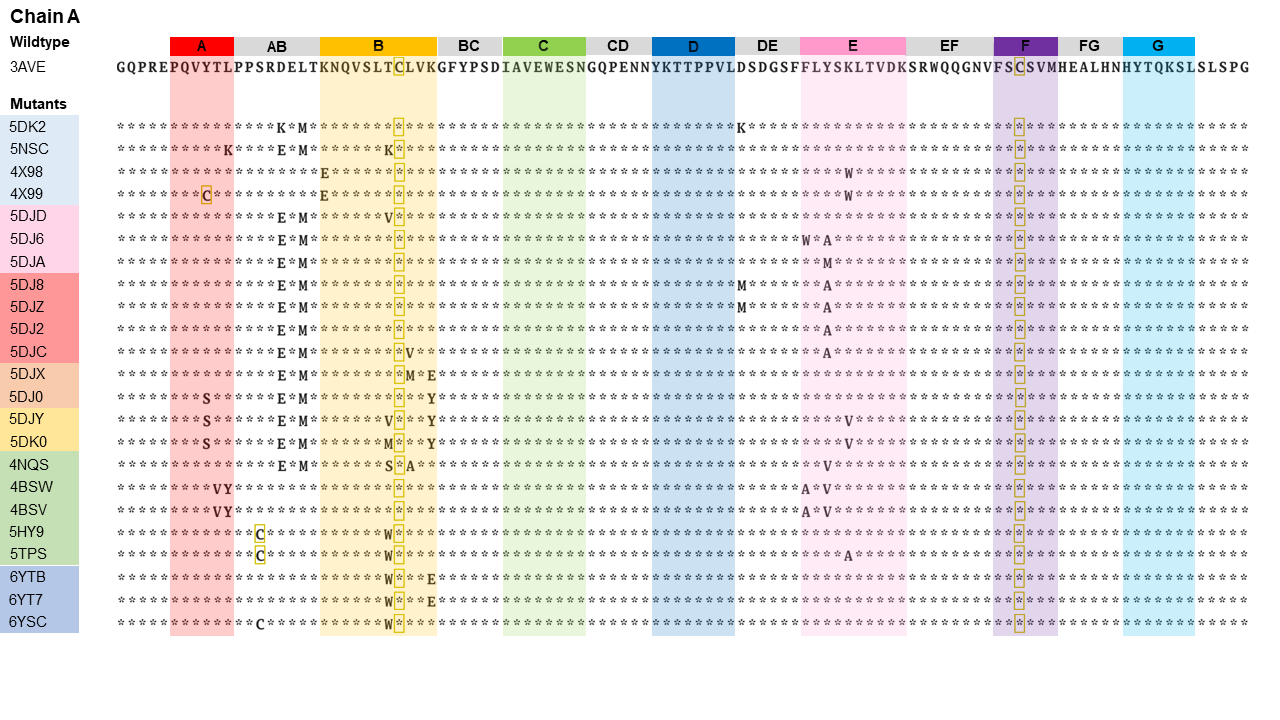

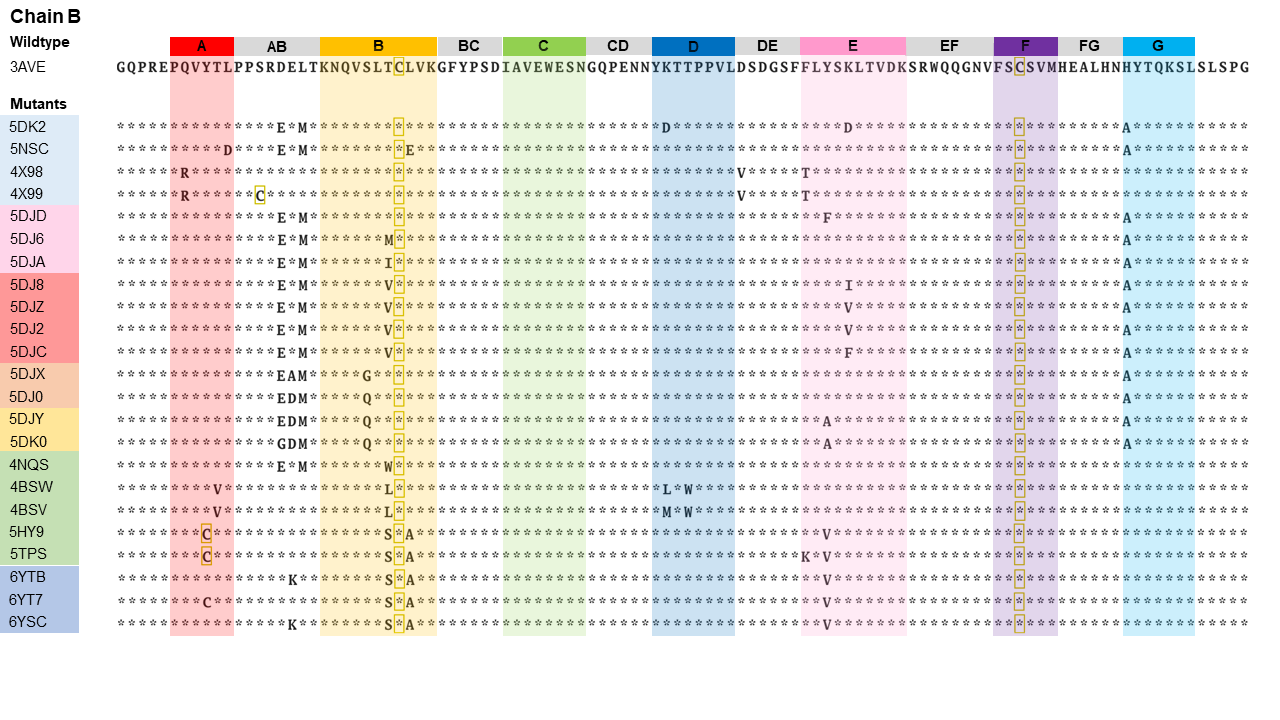


SI Figure S6: Sequence alignment of all investigated C_H_3-C_H_3 dimers. The color-coding refers to the sheet/loop definitions presented in the flareplots. As reference sequence the homodimer (PDB code: 3AVE) has been used.


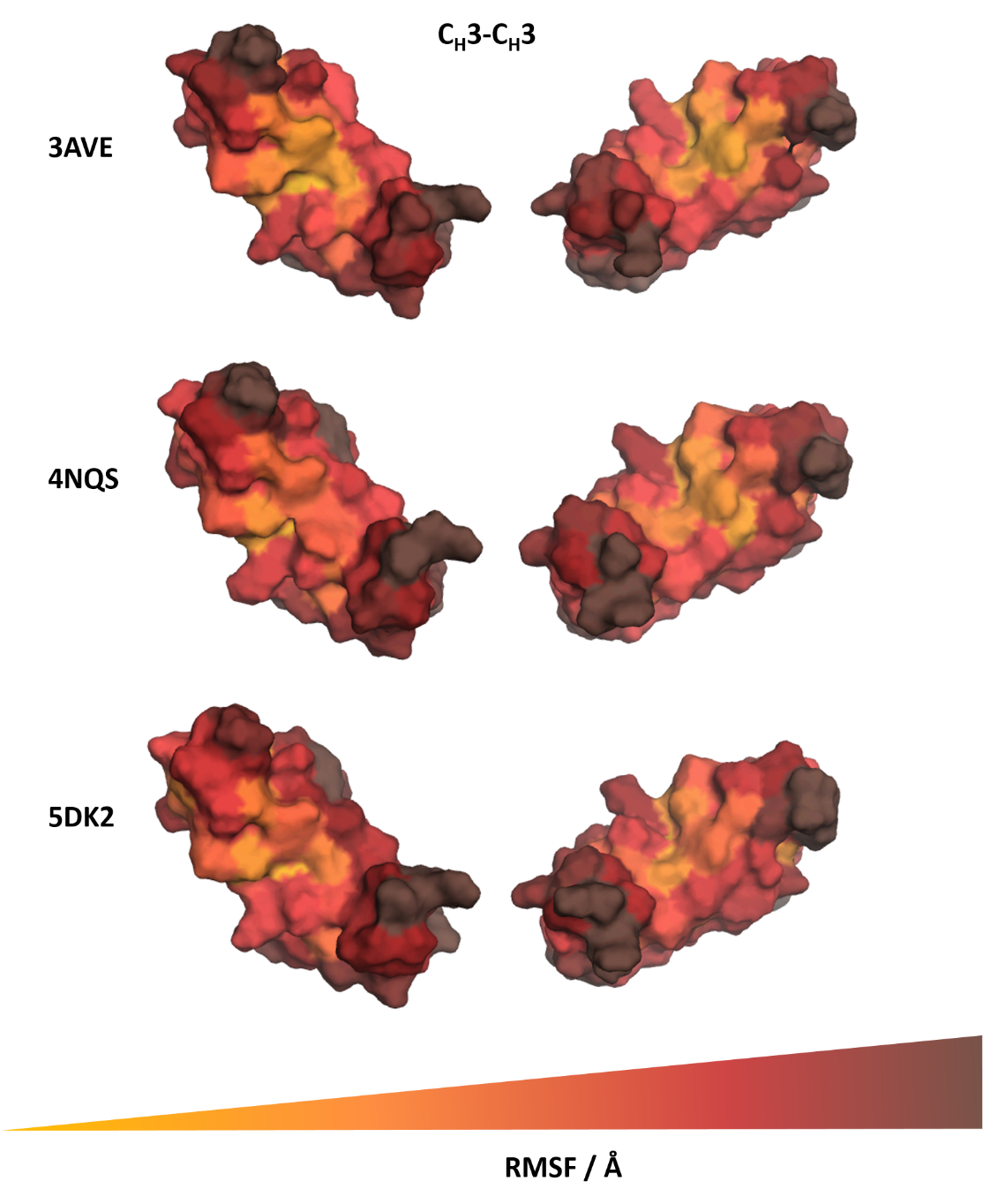


SI Figure S7: Surfaces of different C_H_3-C_H_3 dimers color-coded based on their respective RMSF values (the darker the color (red/brown), the higher is the flexibility).


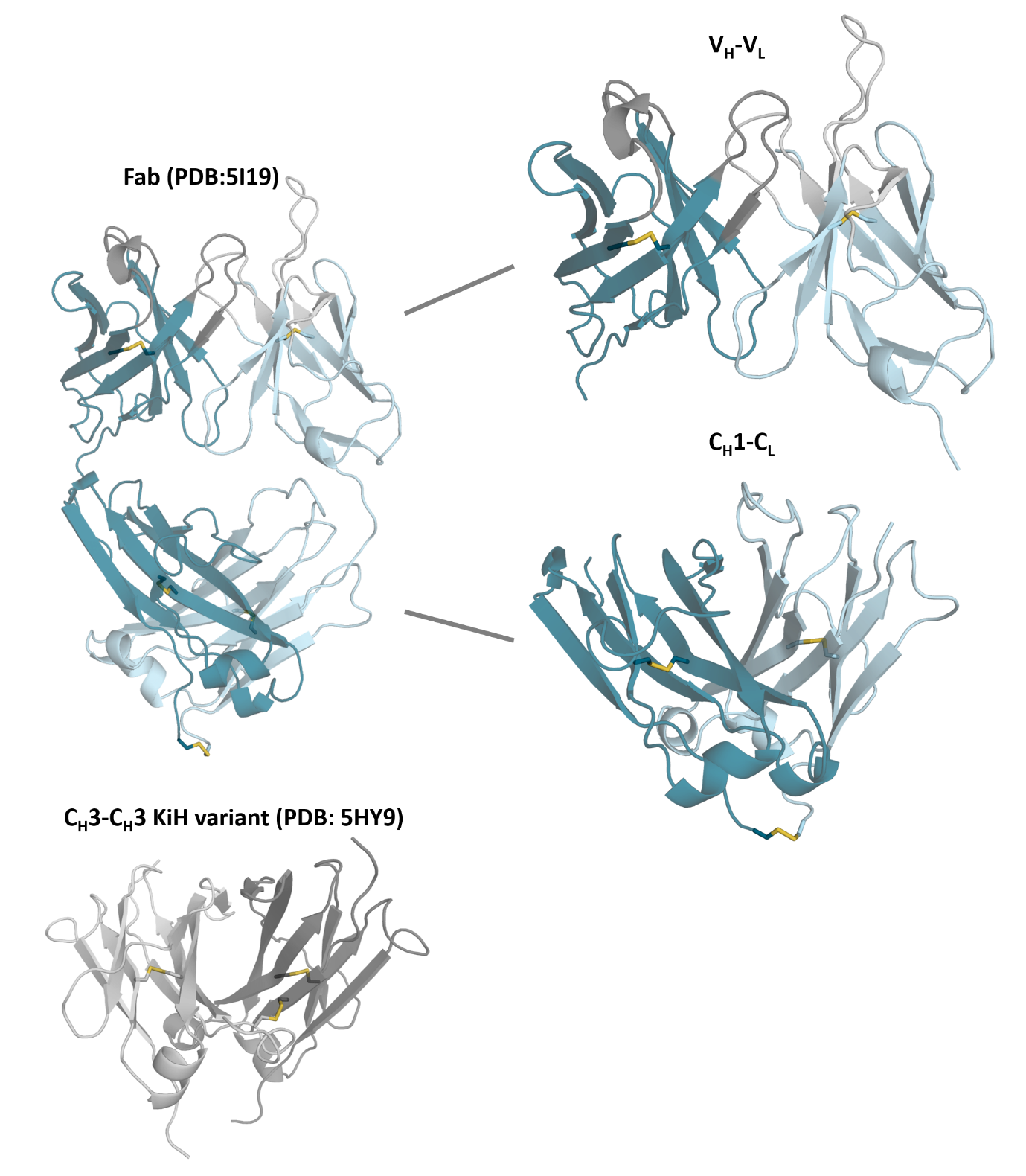
SI Figure S8: Representative structures showing the presence of the intra-and-intermolecular disulfide bridges for the Fab and the C_H_3-C_H_3 structures.

***Parameter file used to perform 1µs of classic molecular dynamics simulations:***

&cntrl

ntb=2

ntp=1,pres0=1.0,taup=2.0

iwrap=1

ioutfm=1

ntx=5, irest=1

cut=8.0

ntc=2,ntf=2

ntt=3,gamma_ln=2.0

tempi=300.0,temp0=300.0

nstlim=500000000,dt=0.002

ntpr=10000,ntwx=10000

/

~
